# Texture Management for Glossy Objects Using Tone Mapping

**DOI:** 10.3390/jimaging8020034

**Published:** 2022-01-30

**Authors:** Ikumi Hirose, Kazuki Nagasawa, Norimichi Tsumura, Shoji Yamamoto

**Affiliations:** 1 Division of Creative Engineering, Graduate School of Science and Engineering, Chiba University, Chiba 263-8522, Japan; nagasawa_kazuki@chiba-u.jp; 2 Graduate School of Engineering, Chiba University, Chiba 263-8522, Japan; tsumura@faculty.chiba-u.jp; 3 Tokyo Metropolitan College of Industrial Technology, Tokyo 140-0011, Japan; yamasho@g.metro-cit.ac.jp

**Keywords:** tone mapping, color matching, glossy, high dynamic range

## Abstract

In this paper, we proposed a method for matching the color and glossiness of an object between different displays by using tone mapping. Since displays have their own characteristics, such as maximum luminance and gamma characteristics, the color and glossiness of an object when displayed differs from one display to another. The color can be corrected by conventional color matching methods, but the glossiness, which greatly changes the impression of an object, needs to be corrected. Our practical challenge was to use tone mapping to correct the high-luminance part, also referred to as the glossy part, which cannot be fully corrected by color matching. Therefore, we performed color matching and tone mapping using high dynamic range images, which can record a wider range of luminance information as input. In addition, we varied the parameters of the tone-mapping function and the threshold at which the function was applied to study the effect on the object’s appearance. We conducted a subjective evaluation experiment using the series category method on glossy-corrected images generated by applying various functions to each display. As a result, we found that the differences in glossiness between displays could be corrected by selecting the optimal function for each display.

## 1. Introduction

In recent years, due to the spread of new coronavirus infections, there has been a rapid increase in the use of video communication [1,2]. Video communication systems have been introduced in many companies because they can be implemented with ease, using familiar devices without the need to prepare new dedicated devices or systems. Currently, online business negotiations are also used for purchasing cars, and in some cases, users confirm the condition of the car through images and videos without seeing the actual car and then purchase it. However, with current video communication systems, depending on the user’s environment, the actual color and texture may differ from those of the actual object, giving a different impression [3,4]. In particular, the color and texture of an object play a very important role in telemedicine, online business negotiations, and meetings conducted through video communication, and thus, accurate information about patients and products must be conveyed. If the color or texture of an object is different from that viewed by the other party, it may lead to serious problems such as cancellation of the transaction or misdiagnosis. In video communication, one of the possible causes for differences in color and texture is the effect of differences in displays. Since each display has different characteristics, such as maximum luminance, gamma, and color gamut, it is not always the same—even if the same image is displayed.

In this study, we propose a method for matching the color and texture of objects between different displays by using tone mapping. In addition, we focused on glossiness among the different textures. Since glossiness is mainly affected by changes in the maximum luminance of the display showing the object, a display method that does not depend on changes in the viewing environment is required for products for which visibility of color and gloss is important, and for human skin in telemedicine. We analyzed the perception of texture and implemented a method for correcting the glossiness of a real object with gloss.

**Figure 1:**
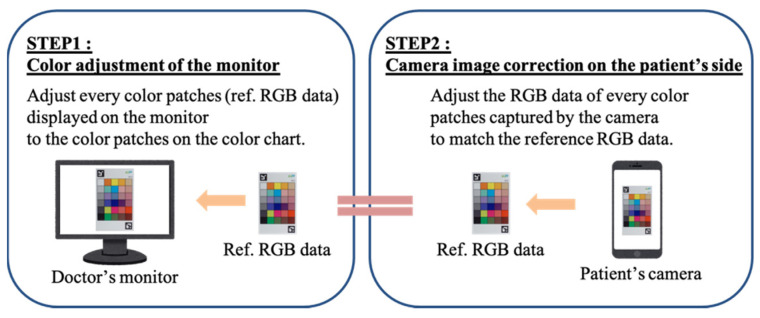
Overview of the automatic color correction method.

For display-independent color reproduction, Takahashi et al. proposed a color reproduction method using a color chart to improve the color quality of telemedicine systems [5]. In their study, based on the color chart, the images sent from the patient side were color corrected and displayed on the doctor’s display to enable accurate diagnosis in telemedicine. The flow of such a system is shown in Figure 1.

With this method, it is first necessary to adjust the RGB values of all the color patches displayed on the display to match those of the actual color patches on the color chart. To adjust the color of the color chart, the XYZ values of each color patch on the color chart are measured using a colorimeter under the same lighting environment as that of the doctor, because humans recognize colors by the three stimulus values XYZ. The monitor side correction is performed with reference to [6]. In addition, from the colorimetric values, the reference RGB values of the color patches for each display are calculated. Color reproduction is achieved by converting the RGB values of each patch in the image captured by the patient to the calculated values.

To transform the captured image, the color chart in the image is first detected using AR markers, and the values of each patch in the color chart are averaged. The model is created by multiple regression analysis based on the difference between the average value and the reference RGB value, and the colors in the image are modified by transforming all pixel values based on this model.

## 2. Materials and Methods

### 2.1. Correction Procedure

In this study, we aim to correct the color reproduction and glossiness of objects between different displays. As a method for reproducing the color of objects, we use color matching, using the color chart described in Section 2. In conventional color matching, processing is based on the color gamut of the color chart; therefore, if saturated areas exist in the image, then processing cannot be performed appropriately. In this case, information from high-luminance areas may be lost and the glossiness may change. Therefore, to achieve a representation that is similar to the real object, we used HDR images, which can retain a wide range of luminance information and glossy information without degradation [7,8]. As an HDR image cannot be displayed on a normal low-dynamic range (LDR) display, it is compressed to a dynamic range that can be displayed on an LDR display by applying tone mapping. Many tone mapping methods have been proposed previously, and they can be generally classified into global tone mapping, which compresses the dynamic range by applying a single function to the entire image, and local tone mapping, which applies different functions depending on the contrast of the image [9,10]. In both cases, by applying a tone mapping function to the entire image, the luminance of the high-luminance areas is reduced, whereas that of the low-luminance areas is increased; as such, the entire image can be represented without information loss. However, when correcting the glossiness of an image, reducing the luminance of the high-luminance areas may cause the glossiness of the image to be lost. Therefore, in the proposed method, color matching is performed on the HDR image to accurately reproduce the colors, and then tone mapping is applied only to the glossy part of the object to correct the glossiness between different displays. The reference display is an iPhone7, and the target displays to be matched are an Xperia1 and FireHD8. The target object is an ornamental bell with a glossy surface.

### 2.2. Color Matching for HDR Images

An HDR image is an image that can represent the entire luminance range and color gamut of a real scene as perceived by the human eye. Since it can handle images with a high contrast ratio as the naked eye does, color matching using HDR images as input can reproduce colors while retaining the glossy information of the object in the image.

First, to create a HDR image, we performed HDR synthesis, which is a technique for creating a HDR image by combining the best dynamic range of multiple images taken at different exposure times. In this paper, we used exposure fusion [11]. This method extracts information such as luminance, saturation, and contrast from each image, and combines them according to the weight assigned to each pixel in each image so that only the best part is retained. The images used for HDR synthesis were taken with a digital single-lens camera (SONY ILCE-5100, resolution 6000 × 4000 px, Sony, Minato City, Tokyo, Japan) under the same shooting conditions, except for the exposure time.

The object and the color chart were photographed at the same time to perform color matching. The color chart used is a special color chart that does not bend under normal use and is printed on a special matte paper using an industrial inkjet printer, like the one used in [5].

**Figure 2:**
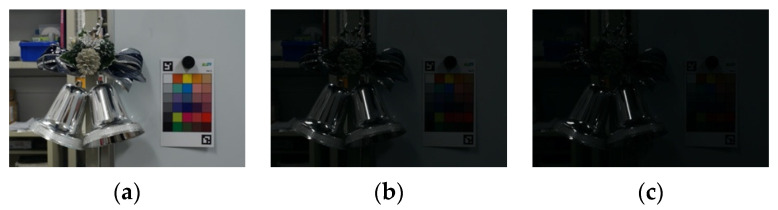
Images with different exposure times. (**a**) 1/8 s; (**b**) 1/60 s; (**c**) 1/125 s.

**Figure 3:**
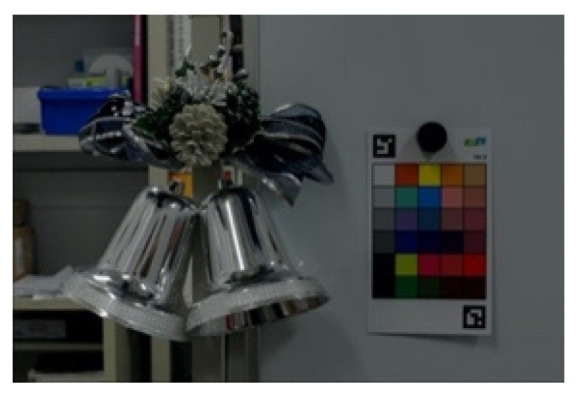
Generated high dynamic range image.

The three images taken are shown in Figure 2 and the HDR image based on them is shown in Figure 3. The exposure time was 1/8 s, 1/60 s and 1/125 s, respectively, and the images with a relatively short exposure time and without saturated glossy areas were used. The resulting HDR image can hold a wider range of luminance than a normal LDR image, so it is believed that the information in the glossy areas was recorded.

**Figure 4:**
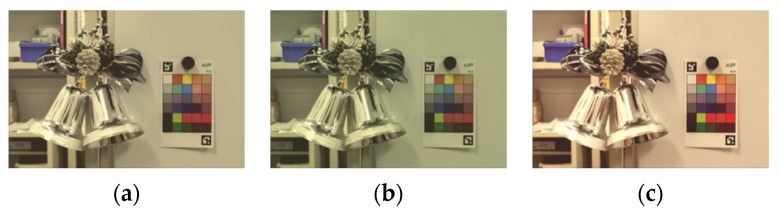
Color matching images. (**a**) iPhone7; (**b**) Xperia1; (**c**) FireHD8.

Using the generated HDR image as input, we performed color matching for each target display based on the method described in Section 1. We used a color luminance meter CS-100A from KONICA MINOLTA (Chiyoda City, Tokyo, Japan) to measure the color of the color charts and displays. The color matching results for the iPhone7, Xperia1, and FireHD8 are shown in Figure 4.

**Figure 5:**
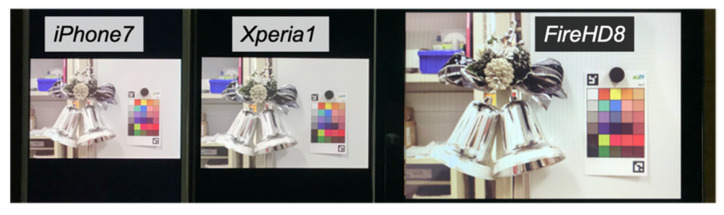
Color matching images displayed on each display.

**Figure 6:**
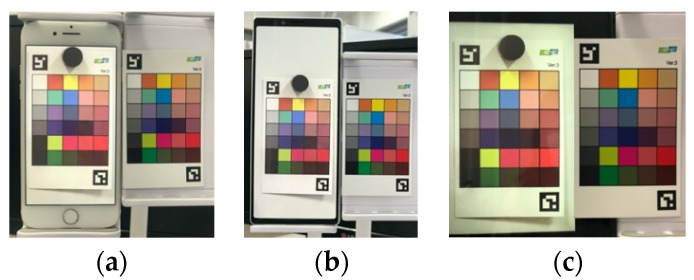
Comparison of the actual color chart and that on each display. (**a**) iPhone7; (**b**) Xperia1; (**c**) FireHD8.

Figure 5 shows the color matching images displayed on each display, and Figure 6a–c compares the actual color chart with the color chart on each display.

Comparing Figure 6a–c, we can see that the colors appear to vary with the different displays, but when these images are displayed on each display, as shown in Figure 5, the color differences between the displays almost disappear visually, indicating that the color matching is executed accurately. In addition, as shown in Figure 6, the accuracy of the color matching is sufficient when compared with the actual color chart.

### 2.3. Generation of Glossy-Corrected Images

In the tone-mapping process, since the dynamic range in a color image mainly affects the luminance information, it is necessary to keep the chromaticity information consistent to create a visually natural image. Therefore, we converted images from the RGB color space to the YUV color space to separate luminance and chromaticity information. Studies based on cone responses have shown that the human eye is sensitive to changes in luminance but insensitive to changes in color, since contrast sensitivity involves a higher resolution than color contrast sensitivity [12]. Hence, in the YUV color space, chromaticity is suppressed, and a wide bandwidth as well as the number of bits are allocated to luminance. By processing only luminance component Y in the YUV color space, we can adjust the dynamic range without affecting the chromaticity information.

**Figure 7:**
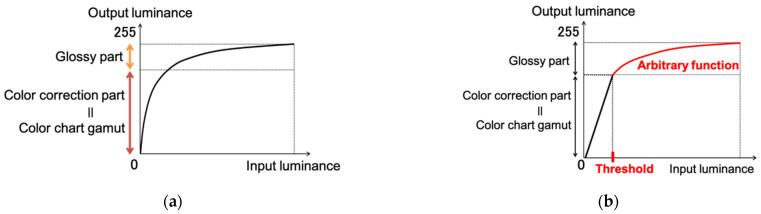
Tone mapping function. (**a**) Conventional tone mapping function; (**b**) Proposed tone mapping function.

If the conventional tone mapping function shown in Figure 7a is applied, then nonlinear processing is also applied to the area where colors are corrected (i.e., the area that corresponds to the color gamut of the color chart), and changes may occur in the area where colors are corrected by color matching. Therefore, to compress the dynamic range while preserving the results of the color correction by color matching, we use a tone mapping function, as shown in Figure 7b. The color-corrected portion is compressed linearly, and an arbitrary tone curve is applied only to the glossy portion, which is outside the gamut of the color chart, i.e., where white blur occurs, to correct the glossiness of the object. Since the color matching method used in this study is based on a color chart, the color correction can be applied only within the color gamut of the color chart used.

However, the glossy part of the object was very bright and did not fit within the color gamut of the color chart, so this method was effective. For the arbitrary functions in the tone curve, we applied the gamma function and the logarithmic function, which are also used in general tone mapping. The gamma function adjusts the distribution of tones and maintains the high and low luminance areas while changing the midpoints to be brighter, whereas the logarithmic function has the property of smooth correction for both ends.

**Figure 8:**
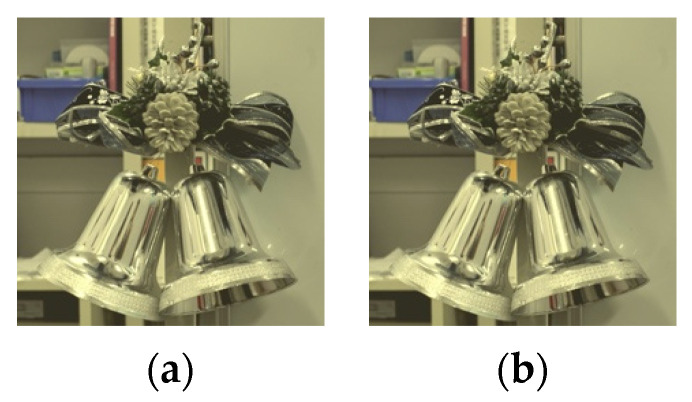
Images with logarithmic function applied (Xperia1). (**a**) threshold: 0.9 parameter: 1.5; (**b**) threshold: 0.9 parameter: 3.5.

**Figure 9:**
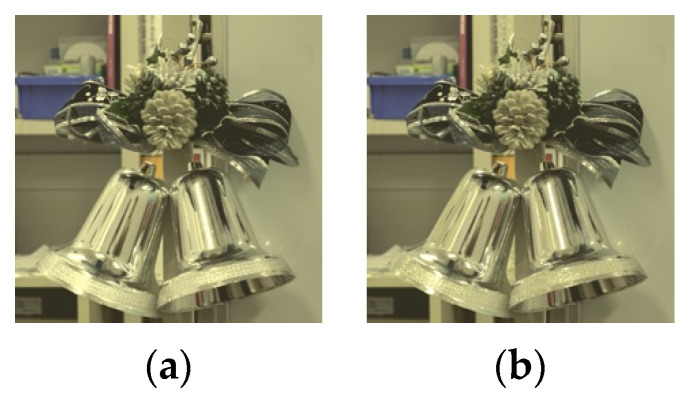
Images with gamma function applied (Xperia1). (**a**) threshold:0.9 parameter: 1.5; (**b**) threshold: 0.8 parameter: 0.5.

**Figure 10:**
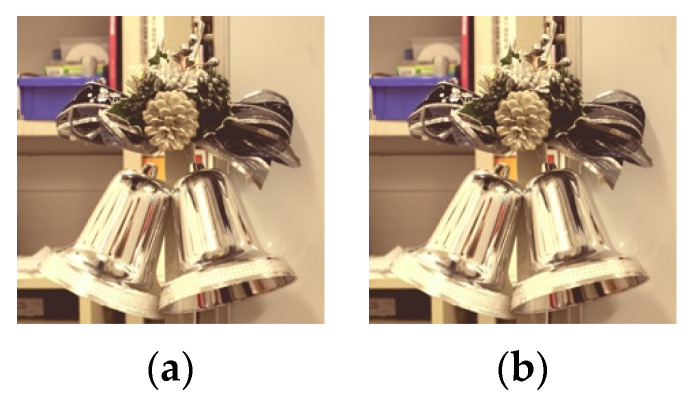
Images with logarithmic function applied (FireHD8). (**a**) threshold: 0.9 parameter: 1.5; (**b**) threshold: 0.9 parameter: 3.5.

**Figure 11:**
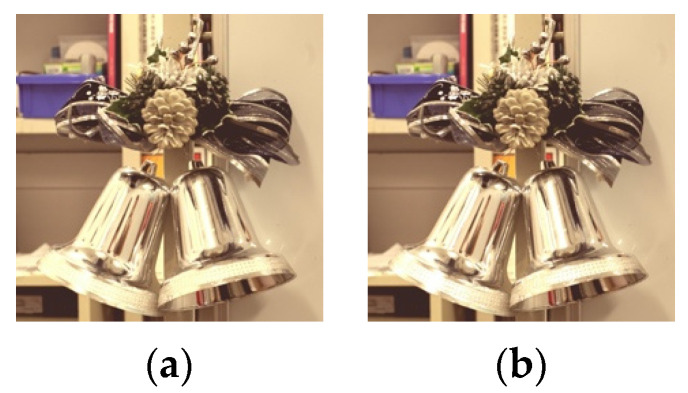
Images with gamma function applied (FireHD8). (**a**) threshold: 0.9 parameter: 1.5; (**b**) threshold:0.9 parameter: 0.5.

The tone-mapping function applied to the image is shown in Equations (1) and (2):

(1) $$Y^{\prime }=Y^{1/\gamma }$$

(2) $$Y^{\prime }=\frac{\log\left(1+Y*\alpha \right)}{\log\left(1+\alpha \right)}$$

where *Y*′ is the luminance transformed by tone mapping; *Y* is the luminance of the original image; and α and γ are the parameters of each function. By varying the parameters in the function and the threshold for applying the function to the luminance component *Y*, multiple glossy corrected images are generated for each display. Figure 8, Figure 9, Figure 10 and Figure 11 show a part of the glossy-corrected image obtained by the processing series.

## 3. Results

The proposed method in the previous section can generate several images with corrected glossiness by applying various tone mapping functions. Here, it is necessary to select the optimal glossy-corrected image for each display, because the appearance of images transformed using the same function varies depending on the characteristics of the display. Therefore, in this section, we execute a subjective evaluation experiment and examine the highly rated images in terms of their gloss correction.

### 3.1. Series Category Method

The series categorical method [13,14] is one method for converting the subjective evaluation values obtained from a group of stimuli into interval scales.

In the subjective evaluation experiment, the subject judges which category the given stimulus falls into, and the weighted average value is obtained from the frequency of each category, and the subject is ranked. However, since the psychological distances between the categories are not necessarily evenly spaced, the calculated values can only determine the direction of the ranking. Therefore, by using the serial categorical method to determine the psychological distance between each category, the obtained weighted mean and standard deviation become the values that directly constitute the interval scale and can be utilized for positioning the evaluation target and determining the boundary value of the category.

The series categorical method is used based on the following assumptions:

(a) Large and small relationships are defined between categories.
(b) The boundaries of the categories are clearly defined.
(c) The evaluation results obtained from the evaluation of stimulus *S*4 are normally distributed on the continuum.
(d) The standard deviations that indicate the flicker of the grading results for each stimulus *S*4 have approximately equal values.

By making the above assumptions, the width of the category and the mean value, and so on, can be determined. In this method, the psychological interval scale values for many objects can be obtained with relatively little effort. It is possible to obtain psychological interval scale values for a considerable number of objects with relatively little effort.

### 3.2. Procedure of Subjective Evaluation

**Figure 12:**
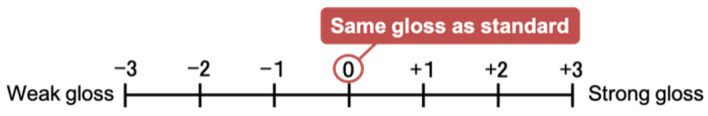
Rating scales.

To select the best tone mapping function for each display and to verify the effectiveness of the proposed method, we conducted a subjective evaluation experiment. In the method for this experiment, images are displayed on two displays and presented to the subject simultaneously. Then, images with only color matching applied to the reference display, as well as images with color matching and tone mapping applied to the target display, are displayed and evaluated. In this experiment, as mentioned above, the reference display is an iPhone7 and the target displays are an Xperia1 and FireHD8. The evaluation item is the consistency of glossiness compared to the reference, and the evaluation scale is shown in Figure 12; 0 indicates images where the glossiness of the object displayed on the target display is consistent with the reference display, +1, +2, +3 indicates images where the glossiness is stronger than the reference, and −1, −2, −3 indicates images where the glossiness is weaker than the reference. The observers were asked to rate the glossiness of an object displayed on a target display with respect to a reference display on a scale of −3 to 3. We asked the observers to focus on a certain object in the image by instructing them to focus on the bell in the image and evaluate its glossiness. We did not ask the observers to train the perceptual meaning of the scale, and the evaluated values were examined via statistical analysis for this method involving successive categories. Moreover, we did not ask the observers to disregard the color differences remaining after color matching; however, we asked the observer to focus on the appearance of the gloss. The observers assessed the various images and displays randomly for the display devices; however, the order was the same for all subjects. All display settings of the devices were maintained constant during the experiment. Furthermore, the effect of screen brightness was not considered; in fact, it was maintained constant at the maximum brightness, and the brightness correction function was turned off. Similar to many mobile devices, the automatic brightness correction function was used occasionally; we plan to conduct future studies by performing this.

**Figure 13:**
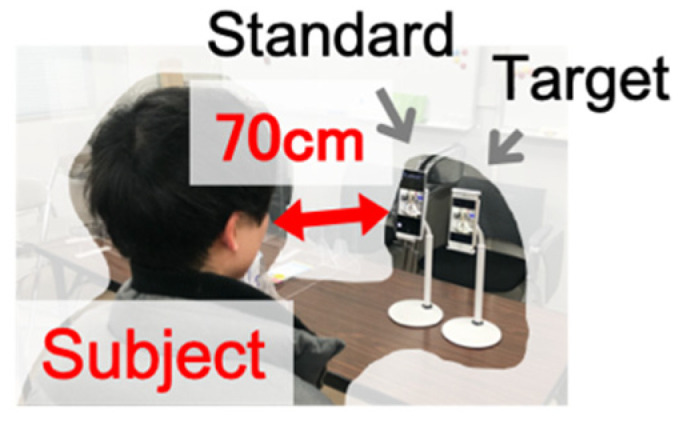
Experimental condition.

This experiment was conducted in the environment shown in Figure 13, with the distance between the subject and the display set at 70 cm. The experimental environment was set to be the same as that used for measuring the brightness of the color chart and the display. The subjects were seven male and female students in their 20 s, and the evaluation time was unlimited. The evaluation targets were 38 images for Xperia1 and 39 images for FireHD8, which were obtained by applying color matching and various tone mapping functions to HDR images. Regarding the characteristics of the three devices, the maximum luminance was 625, 391, and 534 cd/m^2^ for the iPhone7, Xperia1, and FireHD8 displays, respectively.

### 3.3. Results of Evaluation Experiment

**Figure 14:**
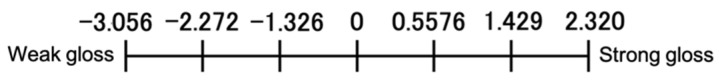
Rating scales after scale construction.

**Figure 15:**
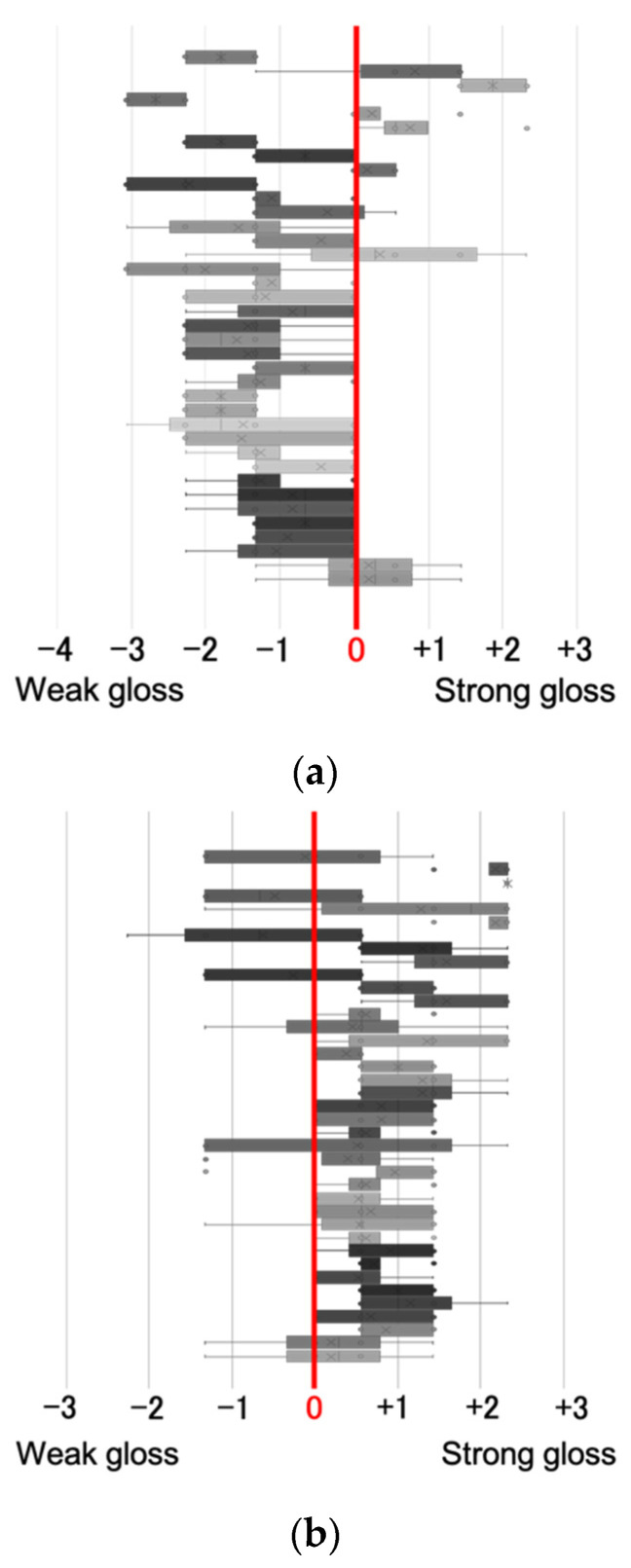
Results of Evaluation Experiment. (**a**) Xperia1; (**b**) FireHD8.

The evaluation values obtained from the experiment were scaled using the serial categorical method, and the results are shown in Figure 14; these values are used in the analysis. Figure 15 shows the results of the subjective evaluation experiment for all images.

**Figure 16:**
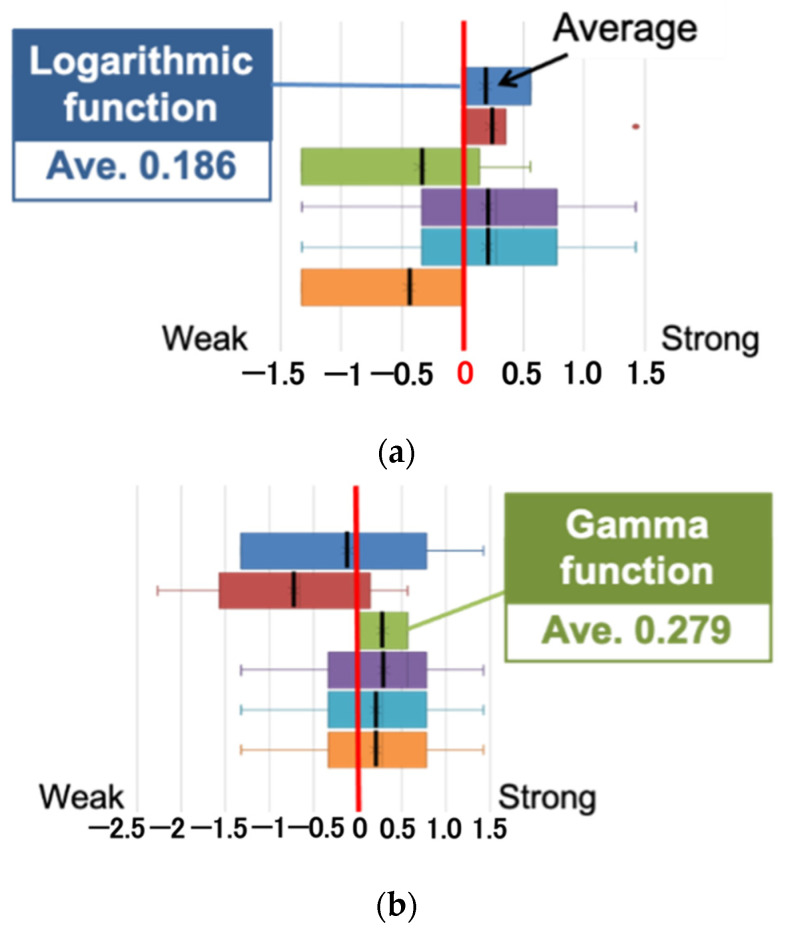
Results of the evaluation experiment. (**a**) Xperia1; (**b**) FireHD8.

**Figure 17:**
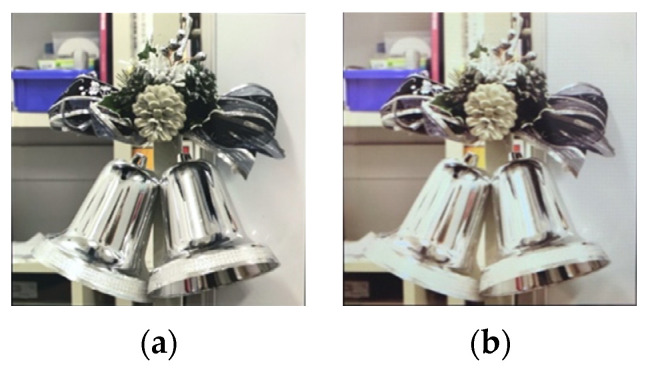
Gloss-corrected image with the best evaluation. (**a**) Xperia1, threshold:0.9, parameter:3.0; (**b**) FireHD8, threshold:0.85, parameter:0.5.

Figure 16 shows the results of the six images with the best evaluation among all images. For the glossiness evaluation in this study, the image with the mean evaluation value closest to 0, without any positive or negative variation among subjects, is the best. The image with the logarithmic function processed with a parameter of 3.0 and a threshold of 0.9 was the one with the mean value closest to 0 on the Xperia1. In this case, the evaluation mean value was 0.186. On the other hand, the image processed with the gamma function with a parameter of 0.5 and a threshold value of 0.85 had a mean value of 0.279, which was the closest to 0 on the FireHD8. The images are shown in Figure 17a,b. The experimental results show that it is possible to correct for glossiness by selecting the optimal tone mapping function that differs for each display.

The model equations for the logarithmic and gamma functions were obtained from a multiple regression analysis of the evaluated values using the statistical analysis software R. The equation for the logarithmic function is shown in Equation (3), and the model equation for the gamma function is shown in Equation (4):

(3) $$G=-8.167a_{1}+1.661a_{2}+0.012L-1.553$$

(4) $$G=-2.441a_{1}+0.237a_{2}+0.015L-5.188$$

where $a_{1}$ is the threshold to apply the function, $a_{2}$ is the parameter in the function, and L is the maximum luminance value of the display. The coefficient of determination *R*^2^ in the multiple regression analysis is the contribution rate that indicates how well the regression equation is applied. In Equation (3), $R^{2}=0.7946$, and in Equation (4), $R^{2}=0.8909$, which means that two equations had strong reliability. From the obtained model equations, we can see that applying a smaller threshold, i.e., a function with a larger slope over a wider area, results in a stronger perception of glossiness.

## 4. Discussion

The effect of tone mapping depends on the parameters of the function, the threshold value, the reflection characteristics of the object, and the characteristics of the display. Therefore, it is necessary to select an appropriate tone mapping function for each object and each display. In this study, we applied several functions to specific objects and selected the best function based on the results obtained from subjective evaluation experiments, but it is impossible to select the best function for other objects or displays. Therefore, it is necessary to develop a method that can automatically select the function that outputs the optimal image according to the object and display and adjust these parameters. In the future, we will conduct experiments with more objects and displays to increase the data and then apply the method to machine learning. We will analyze the correlation between the glossiness evaluation and characteristics such as the maximum luminance of each display, and aim to automatically select the optimal function. In this study, the white point and gamma values, except for the maximum luminance of the display, could not be obtained. Therefore, characteristics associated with the abovementioned values should be obtained and described in the future to present more comprehensive results.

In this subjective evaluation experiment, we assumed that the iPhone7, which is the reference display, had the same glossiness as the real object, and compared it with the target display. However, the glossiness of the actual object and the object on the display must match, so it is necessary to evaluate the actual object in comparison in the future.
